# Notch Ankyrin Repeat Domain Variation Influences Leukemogenesis and *Myc* Transactivation

**DOI:** 10.1371/journal.pone.0025645

**Published:** 2011-10-13

**Authors:** Jon C. Aster, Nick Bodnar, Lanwei Xu, Fredrick Karnell, John M. Milholland, Ivan Maillard, Gavin Histen, Yunsun Nam, Stephen C. Blacklow, Warren S. Pear

**Affiliations:** 1 Department of Pathology, Brigham and Women's Hospital, Boston, Massachusetts, United States of America; 2 Department of Pathology and Lab Medicine, Abramson Family Cancer Research Institute, Institute for Medicine and Engineering, Perelman School of Medicine, University of Pennsylvania, Philadelphia, Pennsylvania, United States of America; 3 Department of Cancer Biology, Dana Farber Cancer Institute, Boston, Massachusetts, United States of America; 4 Department of Biological Chemistry and Molecular Pharmacology, Harvard Medical School, Boston, Massachusetts, United States of America; University of Maastricht (UM), Netherlands

## Abstract

**Background:**

The functional interchangeability of mammalian Notch receptors (Notch1-4) in normal and pathophysiologic contexts such as cancer is unsettled. We used complementary *in vivo*, cell-based and structural analyses to compare the abilities of activated Notch1-4 to support T cell development, induce T cell acute lymphoblastic leukemia/lymphoma (T-ALL), and maintain T-ALL cell growth and survival.

**Principal Findings:**

We find that the activated intracellular domains of Notch1-4 (ICN1-4) all support T cell development in mice and thymic organ culture. However, unlike ICN1-3, ICN4 fails to induce T-cell acute lymphoblastic leukemia/lymphoma (T-ALL) and is unable to rescue the growth of Notch1-dependent T-ALL cell lines. The ICN4 phenotype is mimicked by weak gain-of-function forms of Notch1, suggesting that it stems from a failure to transactivate one or more critical target genes above a necessary threshold. Experiments with chimeric receptors demonstrate that the Notch ankyrin repeat domains differ in their leukemogenic potential, and that this difference correlates with activation of *Myc*, a direct Notch target that has an important role in Notch-associated T-ALL.

**Conclusions/Significance:**

We conclude that the leukemogenic potentials of Notch receptors vary, and that this functional difference stems in part from divergence among the highly conserved ankyrin repeats, which influence the transactivation of specific target genes involved in leukemogenesis.

## Introduction

Notch receptors participate in a highly conserved signaling pathway that regulates cellular differentiation and homeostasis in a dose- and context-dependent fashion (for review, see [1]). Mammals express four different Notch receptors (Notch1-4), large type I transmembrane glycoproteins composed of a series of characteristic structural motifs. Activation of Notch receptors normally depends on two successive types of proteolytic cleavages (for review, see [2]). First, binding of ligands to the extracellular domain of Notch triggers proteolytic cleavage just external to the transmembrane domain by ADAM-type metalloproteases. This creates a truncated membrane-tethered form of Notch that is recognized by the gamma-secretase protease complex. Additional cleavages by gamma-secretase free the intracellular domain of Notch (ICN) from the membrane, allowing it to translocate to the nucleus and form a transcriptional activation complex with the DNA-binding factor CSL and a co-activator protein of the Mastermind-like (MAML) family.

Structural studies have provided a model for the stepwise assembly of the CSL/ICN/MAML transcriptional activation complex [3], [4]. The intracellular portions of Notch1-4 (ICN1-4) contain N-terminal RAM domains, which bind CSL with relatively high affinities [5], [6], [7], and 7 iterated ankyrin repeats. Under physiologic conditions, the RAM domain likely mediates the initial association of ICN and CSL, which enables formation of a CSL∶ANK composite surface that recruits MAMLs, an essential event for transcriptional activation of target genes and subsequent downstream functions [8], [9].

**Figure 1:**
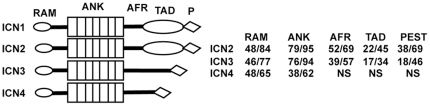
ICN1-4 Sequence Homology. The numbers at right are the %identity/%overall identity and similarity for each indicated domain of ICN2, ICN3, and ICN4 relative to ICN1, based on sequence alignments and homology calls performed by Clustal. Similarity is defined as positions with conservative or semi-conservative substitutions. N.S. indicates no significant homology. Domain boundaries were defined based on ICN1, in which the RAM domain and flanking C-terminal sequences span residues 1761 to 1872; the ankyrin repeat domain (ANK) spans residues 1873 to 2123; an ANK flanking region (AFR) spans residues 2124 to 2204; the transcriptional activation domain spans residues 2205 to 2422 [10]; and the C-terminal domain including the PEST degron spans residues 2423 to 2556.

In line with their critical role in assembly of this complex, ANK domains are the most highly conserved part of ICN1-4, followed by the RAM domains (summarized in Figure 1). In contrast, sequences C-terminal of ANK are substantially more varied. At the far C-termini of ICN1-4 are PEST degron domains that stimulate ICN degradation. Between the ANK and the PEST domains lies the most divergent portion of mammalian ICN1-4. In ICN1 this region includes a strong transcriptional activation domain (TAD), whereas the analogous region of Notch2 appears to contain a weaker TAD [10]. The same portion of Notch3 lacks a conventional TAD, but instead is proposed to interact with an as-of-yet unknown zinc-finger transcription factor that contributes to the activation of specific target genes, such as *Hes5* [11]. Sequences immediately C-terminal of ANK in ICN1 and ICN2 contain phosphorylation sites that may modulate function in response to cytokines [12], [13], [14]. The function of the region between ANK and PEST in ICN4 is unknown.

Although ICN1-4 differ in their transactivation of Notch-responsive reporter genes in transient expression assays [11], [15], most direct comparisons performed to date *in vivo* have not revealed functional differences. Replacement of the last 426 amino acids of Notch2 with the same region of Notch1 (corresponding to the seventh ankyrin repeat, the TAD, and the PEST domain) by gene targeting results in normal mice [16], suggesting that these portions of ICN1 and ICN2 are equivalent functionally. Of relevance to this report, transduced ICN1 or ICN4 both induce human hematopoietic progenitors to undergo T cell development following transplantation into NOD/SCID mice [17].

An important pathophysiologic outcome of ICN overexpression is neoplasia. Retroviral expression of ICN1 in hematolymphoid progenitors is a potent inducer of murine T-ALL [18], and the majority of human and murine T-ALLs harbor gain-of-function mutations in Notch1 (for recent review, see ref. [19]. Feline leukemia viruses that transduce the coding sequences for the RAM and ANK domains of ICN2 accelerate T-ALL development [20], and transgenic LCK-ICN3 mice develop T-ALL with high penetrance and short latency periods [21], indicating that Notch2 and Notch3 also have leukemic potential. Recent deep sequencing studies have identified acquired mutations that result in deletion of the C-terminal PEST domain in 10-15% of human chronic lymphocytic leukemia (CLL) [22], [23], a type of Notch1 mutation originally identified in human T-cell acute lymphoblastic leukemia (T-ALL) [24] that stabilizes ICN1 and enhances the transactivation of target genes in leukemia cells. Conversely, Notch signaling has tumor suppressive effects in the context of squamous epithelium [25], [26], a finding that emphasizes the context-dependent outcome of Notch signaling.

*Notch4* was first identified as a proviral insertion site in murine mammary tumors, and enforced expression of ICN4 contributes to development of adenocarcinoma [27]. However, the transforming abilities of ICN1-4 have not been compared directly *in vivo* in a single cellular context, and other data suggest that ICNs have divergent activities. For example, ICN1 and ICN2 reportedly have opposing effects on the growth of brain tumors [28]. Thus, the physiologic and pathophysiologic interchangeability of ICN1-4 is an open question.

To address this issue, we compared the ability of ICN1-4 to drive T cell development and cause T-ALL *in vivo* and to rescue T cell progenitors from blockade of endogenous Notch signaling in thymic organ culture assays. We find that while ICN1-4 all support T cell development, only ICN1-3 induce T-ALL efficiently. T cell progenitors expressing ICN4 appear to be actively extinguished and disappear by 6 months post-transplantation, a phenotype resembling that caused by “hypoleukemic” weak gain-of-function forms of Notch1 [29]. Further, studies performed with chimeric receptors allowed us to map the structural basis for this difference in leukemogenicity to repeats 2–7 of the ANK domain, which influence the ability of ICN to activate expression of *Myc*, a key Notch target gene implicated in leukemogenesis. These studies demonstrate that the transforming activities of Notch receptors in hematolymphoid progenitors are not equivalent, and that this functional divergence is attributable in part to variation in the highly conserved ANK domains.

## Results

### ICN1-4 Drive T cell Development *in vivo* and Rescue Developing Thymocytes from the Effects of Gamma-Secretase Inhibitors

**Figure 2:**
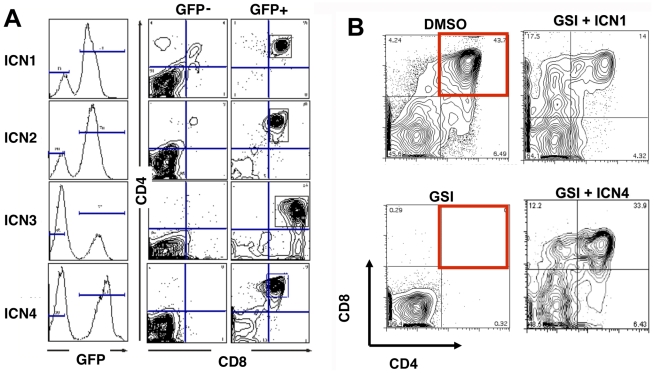
Mammalian ICNs Induce T Cell Development in the Bone Marrow and in Fetal Thymic Organ Cultures. (A) Bone marrow cells were analyzed by flow cytometry on day 24 post-reconstitution of lethally irradiated recipients with marrow transduced with MigRI-ICN1-4. Representative results are shown. (B) Fetal liver hematopoietic progenitors transduced with MigRI, MigRI-ICN1, or MigRI-ICN4 were used to reconstitute irradiated thymic lobes obtained from the same donor animals. Left hand panels: MigRI control lobes treated with vehicle (DMSO) or the gamma-secretase inhibitor (GSI) compound E (1 microM). Right hand panels: MigRI-ICN1 and MigRI-ICN4 lobes treated with compound E (1microM).

When expressed in hematopoietic progenitors, gain-of-function forms of Notch1 cause a CD4^+^CD8^+^ double-positive (DP) T cell population to appear in the bone marrow by day 24 post-bone marrow transplant (BMT) [18]. To begin to compare the activities of ICN1-4 in hematopoietic cells, we transduced bone marrow progenitors with MigRI retroviruses of equal titer, and used these cells to reconstitute syngeneic recipient animals. On day 24 post-BMT, the marrow of all ICN1-4 animals contained an abnormal GFP^+^ DP T cell population, whereas DP T cells were absent from the GFP^-^ bone marrow cell populations of ICN1-4 animals (Figure 2A), as well as MigRI control animals (data not shown). Thus, ICN1-4 all drive ectopic T cell development from bone marrow progenitors.

To further study the interchangeability of ICN1-4 in developing T cells, we compared the ability of ICN1-4 to rescue T cell development in thymic organ cultures treated with compound E, a potent gamma-secretase inhibitor (GSI) that blocks T cell development at the CD4^−^CD8^−^ double negative (DN) 3a stage by inhibiting ICN1 production. In experiments conducted with transduced fetal liver hematopoietic progenitors, ICN1-4 all rescued DP T cell development in the presence of GSI (Figure 2B and data not shown), indicating that each can induce intrathymic T cell development.

### ICN4 does not Induce T-ALL or Support the Growth of Notch1-Dependent T-ALL Cells

When activated Notch isoforms are expressed in bone marrow progenitors, the appearance of circulating DP T cells is usually a harbinger of the subsequent lethal T-ALL [18]. Mice receiving ICN1-4 transduced progenitors uniformly developed circulating GFP+ DP T cells by day 21 post-transplant. By day 124 post-transplant, all of the ICN1, ICN2, and ICN3 animals developed T-ALL, but ICN4 animals remained healthy and did not develop leukemia (summarized in Table 1). At necropsy, ICN1-3 animals showed widespread involvement of tissues by leukemic blasts, which had immunophenotypes consistent with immature T cells (data not shown). These leukemias were readily transplantable to secondary recipients (summarized in Table 1).

**Table 1:** Summary of Transplant Experiments Performed with Bone Marrow Cells Transduced with ICN1-4.

	ICN1	ICN2	ICN3	ICN4
# of Mice Analyzed	30	24	24	28
**Mean WBC (×10^3^ cells/mm^3^)** *				
Day 21	21+/−3	20+/−2	20+/−3	15+/−5
Day 60	62+/−5	50+/−3	69+/−2	18+/−2
Day 124	94+/−9	110+/−6	86+/−7	19+/−2
**PB GFP^+^ CD4^+^/CD8^+^ Cells (%)** *				
Day 21	26+/−3	20+/−3	22+/−2	14+/−2
Day 60	60+/−3	50+/−4	53+/−3	<1
Day 124	92+/−4	87+/−4	82+/−5	<1
**Leukemia Induction in Secondary Recipients** **				
	Yes	Yes	Yes	No

*+/− 1 S.D.

**As assessed by secondary transfer of GFP^+^ CD4^+^/CD8^+^ bone marrow cells obtained on day 21 post-transplant.

WBC, white blood cell count; PB, peripheral blood.

**Figure 3:**
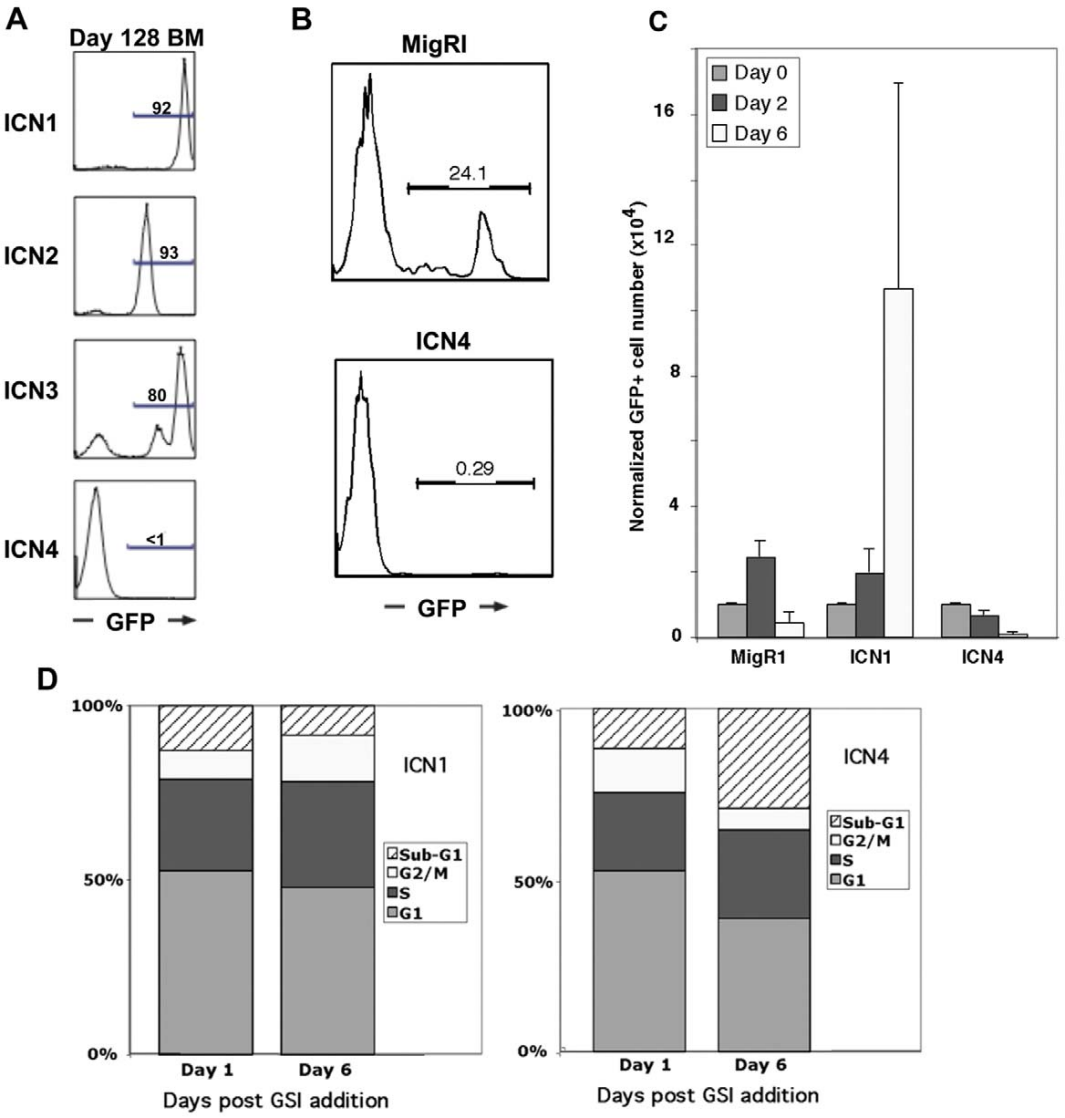
ICN4 does not Induce T-ALL or Rescue Notch-1-Dependent T-ALL Cells. (A) GFP+ marrow populations in mice on day 128 post-reconstitution with marrow progenitors transduced with MigRI-ICN1-4. (B) GFP+ Lin-/Sca1-/c-Kit+ (LSK) marrow populations in mice on d180 post-reconstitution with marrow progenitors transduced with MigRI or MigRI-ICN4. (C) T6E transduced with empty MigRI virus or MigRI-ICN1 or -ICN4 viruses were treated with the gamma-secretase inhibitor (GSI) compound E (1 microM) and monitored for growth. (D) DNA content histograms of T6E cells transduced with ICN1 or ICN4 and treated with compound E (1 microM) for the indicated times.

A clue to the basis for this distinction between ICN1-3 and ICN4 was evident in analyses of bone marrow on day 128 post-transplant. By this time point in ICN4 animals GFP^+^ cells were markedly decreased in number, whereas the GFP^+^ DP T cell populations in ICN1-3 animals continued to expand as T-ALL developed (Figure 3A). In contrast to ICN4 animals, GFP^+^ cells persisted indefinitely in MigRI control animals (data not shown), suggesting that ICN4 suppresses or extinguishes cells with long-term self-renewal capacity. Consistent with this possibility, by 180 days post-transplant ICN4 animals lacked GFP^+^ Lin^−^Sca1^−^c-Kit^+^ (LSK) bone marrow cells, a population that contains cells with long-term self-renewing capacity [30], whereas a persistent GFP^+^ LSK cell population was detectable in MigRI control animals (Figure 3B).

LSK populations in ICN1-3 animals could not be studied because of the development of T-ALL. Of note, however, in prior work we observed that expression of weak gain-of-function non-leukemogenic Notch1 alleles in murine HSCs resulted a burst of T cell development followed by loss of GFP-positivity in the hematolymphoid compartment and no T-ALL development [29], [31], a phenotype closely resembling that produced by ICN4. In addition, expression of ICN1 in Rag1-/- HSCs, which do not develop T-ALL due to defects in lymphoid development, also depletes the LSK population (data not shown). Thus, ICN4 and other forms of activated Notch appear to deplete LSK populations in reconstituted mice, possibly by inducing differentiation at the expense of maintenance of multipotent progenitors.

T-ALL cell lines derived from Notch1-induced T-ALLs require persistent Notch signaling for growth and survival. The murine T-ALL cell line T6E [32] expresses a membrane tethered form of Notch1 that requires gamma-secretase cleavage for activation and can be rescued from GSI-induced apoptosis by retroviral transduction of ICN1, which lies downstream of the GSI blockade. ICN1 rescued T6E cells from GSI induced growth arrest and apoptosis, whereas ICN4 did not (Fig. 3C and 3D). Thus, ICN4 is also incapable of maintaining the growth of established Notch-dependent T-ALL cells.

### ANK Domains Determine Notch Receptor Leukemogenicity

The modular nature of Notch intracellular domains and the availability of a high-resolution structure of the ANK domain of ICN1 [3], [33] facilitated construction of chimeric ICNs, which were used to investigate the structural basis for the functional differences between leukemogenic ICNs and ICN4. Because the greatest variation among ICNs is in the region C-terminal of ANK (Figure 1), we anticipated that addition of this region of ICN1 to the RAM-ANK domain of ICN4 would create a leukemogenic chimera. However, the resulting chimeric polypeptide, ICN4/ICN1-CT, created by joining of the RAM-ANK domain of ICN4 to the C-terminus of ICN1 at position J3 (Figure S1), produced an “ICN4” phenotype characterized by the transient appearance of DP GFP+ T cells in the peripheral blood by day 24 post-transplantation and the absence of leukemia (data not shown).

Because the RAM domain of ICN1 is not essential for leukemogenesis [18], we next focused on ANK, which is required for all known physiologic and pathophysiologic Notch functions.

### Biophysical differences among the recombinant ANK domains are minimal

Recombinant human ANK1-4 proteins exhibit highly similar circular dichroism spectra (Figure S2A). The programs CONTINLL [34], SELCON3 [35], and K2D [36] on the Dichroweb server [37], yield estimates of 36–40% alpha helix for all four molecules. Moreover, there are minimal differences in the thermal stability of the four ANK domains, with Tm values ranging from 46–50°C for the four proteins: (ANK1 T_m_ = 50°C; ANK2 T_m_ = 47°C; ANK3 T_m_ = 46°C; ANK4 T_m_ = 50°C; Figure S2B). These data show that the variations in primary sequence observed in the ANK family do not translate to substantial heterogeneity in secondary structure or domain stability. However, a comparison of ANK1-4 surfaces based on the ANK1 crystal structure [3], [33] predicts that while the contacts involved in CSL/ANK/MAML ternary complex formation are highly conserved, surfaces on the convex faces of ANK domains opposite CSL/MAML contacts are more varied (Figure S3), raising the possibility of functional divergence among the four ANK domains.

**Figure 4:**
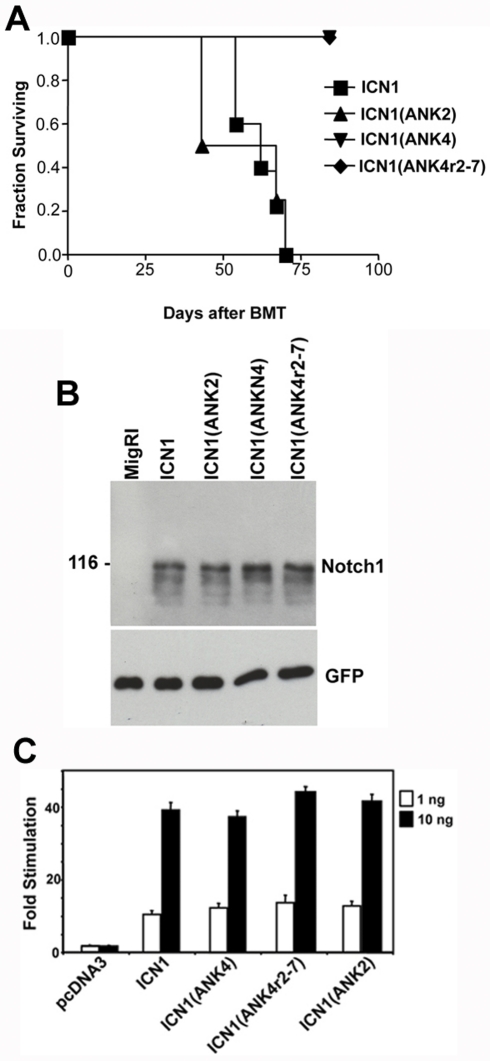
Chimeric ICN Polypeptides Containing the Notch4 Ankyrin Repeats Fail to Induce T-ALL, Despite Comparable Activation of a Notch Reporter Gene. (A) Kaplan-Meier curves for mice reconstituted with bone marrow progenitors expressing ICN1, ICN1(ANK2), ICN1(ANK4), or ICN1(ANK4r2-7). (B) Western blot analysis of U2OS cells transduced with empty MigRI virus or MigRI viruses carrying cDNAs for the indicated ICN polypeptides. After normalization on GFP expression, lysates were analyzed on Western blots stained with either a rabbit polyclonal antiserum specific for the TAD of Notch1 or a monoclonal antibody specific for GFP. (C) U2OS cells grown in 24-well dishes were co-transfected with 1 ng or 10 ng of pcDNA3 plasmids encoding the indicated forms of ICN, an internal control *Renilla* luciferase plasmid, and an artificial Notch-responsive firefly luciferase reporter gene plasmid. Luciferase activities were determined in triplicate 44–48 hr post-transfection. Fold stimulations represent the normalized firefly luciferase activity relative to that of the empty vector control, which is arbitrarily given a value of 1. Error bars represent 1 standard deviation. ICN1(ANK2), ICN1 backbone containing the ANK domain of Notch2; ICN1(ANK4), ICN1 backbone containing the ANK domain of Notch4; ICN1(ANK4r2-7), ICN1 backbone containing ANK repeats 2–7 of Notch4.

Guided by these structural insights, we constructed an ICN1(ANK4) chimera by swapping the ANK of NOTCH4 into the ICN1 backbone at positions J1 and J3 (Figure S1). The resulting ICN1(ANK4) polypeptide produced an “ICN4” phenotype in mice (Figure 4A and Table 2), indicating that the ANK domain of ICN4 is limited in its capacity to support leukemia induction. In contrast, ICN chimeras in which the ANK domains of ICN2 or ICN3 were swapped into ICN1 at positions J1 and J3 were fully leukemogenic (Figure 4A; summarized in Table 2).

**Table 2:** Summary of Transplant Experiments with Chimeric ICNs.

Construct	# of Mice	# with DP T Cells	# of T-ALLs	Mean Onset (days)	Onset Range (days)	Mean WBC (10^3^/mm^3^)	WBC range (10^3^/mm^3^)	Mean spleen weight (mg)	Spleen weight range (mg)
ICN1	5	5	5	62	54–70	140	29–216	0.75	0.52–0.85
ICN1(ANK2)	4	4	4	55	43–70	113	29–176	1.17	0.96–1.23
ICN1(ANK3)	3	3	3	56	43–63	161	24–320	1.17	0.4–1.26
ICN1(ANK4)	4	4	0	N/A	N/A	20.5	19–23	N/A	N/A
ICN1(ANK4r2-7)	4	4	0	N/A	N/A	20	16–23	N/A	N/A

WBC = white blood cell count; DP T cells = CD4+/CD8+ GFP+ T cells in peripheral blood on day 24 post bone marrow transplant; onset = onset of T-ALL, defined as the appearance of symptoms (lassitude, scruffiness), physical signs (organomegaly), and an increasing number of GFP+ DP cells in the peripheral blood.

To assess whether this functional distinction stemmed from differences in protein quantity, we compared the levels of leukemogenic ICN1 and ICN1(ANK2) and non-leukemogenic ICN1(ANK4) chimeric polypeptides in cells transduced with retroviruses of equal titer. Western blots stained with an antibody specific for a common epitope located in the TAD of Notch1 revealed that these forms of ICN were expressed at equal levels (Figure 4B), suggesting that the variation in leukemogenecity stems from qualitative differences. In parallel, we noted that these ICNs activated a sensitive Notch reporter gene to a similar degree (Figure 4C). Together, these findings suggested that the differences in leukemogenic activity stem from qualitative differences in the ability of different ANKs to activate genomic target genes.

Additional experiments were then conducted to further define the structural variation responsible for the observed functional differences. The greatest structural divergence among the ANKs is in the first repeat, which is partially unstructured [3], [33], [38]. We therefore constructed and tested an ICN1(ANK4r2-7) chimera created by swapping ankyrin repeats 2–7 of ICN4 into the backbone of ICN1 at positions J2 and J3 (Figure S1). This chimera also produced an “ICN4” phenotype in mice (Figure 4A; summarized in Table 2).

**Figure 5:**
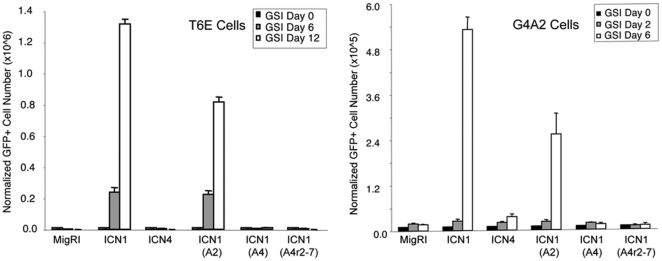
Non-Leukemogenic ICN Chimeras are Defective in Growth Rescue of “Notch1-Addicted” T-ALL Cells. The Notch1-dependent murine T-ALL cell lines T6E and G4A2 were transduced with empty MigRI virus or the indicated forms of ICN and monitored for growth in the presence of the gamma-secretase inhibitor (GSI) compound E (1 microM). ICN1(A2), ICN1 backbone containing the ANK domain of Notch2; ICN1(A4), ICN1 backbone containing the ANK domain of Notch4; ICN1(A4r2-7), ICN1 backbone containing ANK repeats 2–7 of Notch4.

We also tested the contribution of the ANK domain to the growth maintenance of two Notch1 “addicted” GSI-sensitive murine T-ALL cell lines, T6E and G4A4. These results correlated with leukemia induction, as a chimeric ICN containing the leukemogenic ANK of Notch2 supported T-ALL cell growth and survival, whereas chimeric ICNs containing Notch4 ANK or Notch4 ANK repeats 2–7 did not (Figure 5).

### ICN Leukemogenecity Correlates with Transactivation of *Myc*

**Figure 6:**
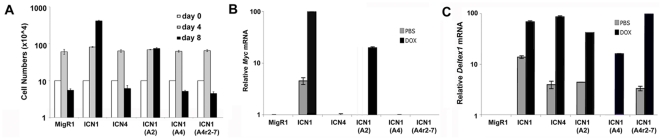
Non-Leukemogenic Forms of ICN Fail to Rescue 8946 Cell Line Growth and do not Transactivate *Myc*. (A) Comparison of rescue of 8946 cells by various ICN polypeptides following doxycycline-mediated withdrawal of *MYC*. 8946 cells were transduced with MigRI viruses, sorted for GFP positivity, and then treated with doxycycline (20 microgram/ml) for up to 8 days. Cell numbers at each time point are shown. (B, C) 8946 cells transduced as in (A) were harvested after 24 hr of treatment with doxycycline (20 microgram/ml) or vehicle (PBS). Levels of *Myc* (B) and *Deltex1* (C) mRNAs were determined by qRT-PCR.

*Myc* is a direct Notch target gene that contributes to leukemogenesis [39], [40], [41], and the failure of certain ICNs to induce T-ALL or maintain T-ALL cell growth might therefore stem from differing abilities to transactivate *Myc*. This idea was tested using the 8946 T-ALL cell line, which was derived from a murine T-ALL induced with a “doxycycline-off” human *MYC* transgene. 8946 cells are rescued from doxycycline treatment by transduction of activated Notch1 [39], which induces expression of endogenous murine *Myc*. We observed that leukemogenic forms of ICN (ICN1 and ICN1(ANK2) rescued the growth of 8946 cells in the presence of doxycycline, whereas non-leukemogenic ICNs (ICN4, ICN1(ANK4), and ICN1(ANK4r2-7)) did not (Figure 6A). None of the ICNs had any effect on the growth of 8946 cells in the absence of doxycycline (Figure S4). Growth rescue correlated with transactivation of the endogenous murine *Myc* gene, as ICN1 and ICN1(ANK2), but not ICN4 or ICN1(ANK4r2-7), induced *Myc* expression (Figure 6B). We also noted that upregulation of endogenous *Myc* by ICN1 and ICN1(ANK2) was blunted by expression of transgenic *MYC* (Figure 6B). Transgenic or translocation-mediated overexpression of *Myc* leads to autorepression of intact endogenous *Myc* loci [42], [43] through a mechanism that appears to involve polycomb family repressors [44]; it may be that autorepression also attenuates ICN transactivation of endogenous *Myc* in 8946 cells. The inability of non-leukemogenic forms of ICN to upregulate *Myc* did not reflect a general defect in transactivation of genomic Notch target genes, as all ICNs tested upregulated *Deltex1* (Figure 6C), and showed varying abilities to upregulate other Notch1 target genes, such as *Ptcra* (pre-T cell receptor-alpha), *CD25*, and *Notch3* (Figure S5). Of note, none of the Notch target genes were expressed in the basal state in 8946 cells and, like *Myc*, showed varying degrees of suppressed responsiveness in the presence transgenic human Myc. Thus, in the *MYC*-induced 8946 cell line there appears to be no selective pressure for expression of Notch target genes, and indeed enforced Myc expression may be associated with epigenetic changes that negate Notch's ability to upregulate its targets.

## Discussion

**Figure 7:**
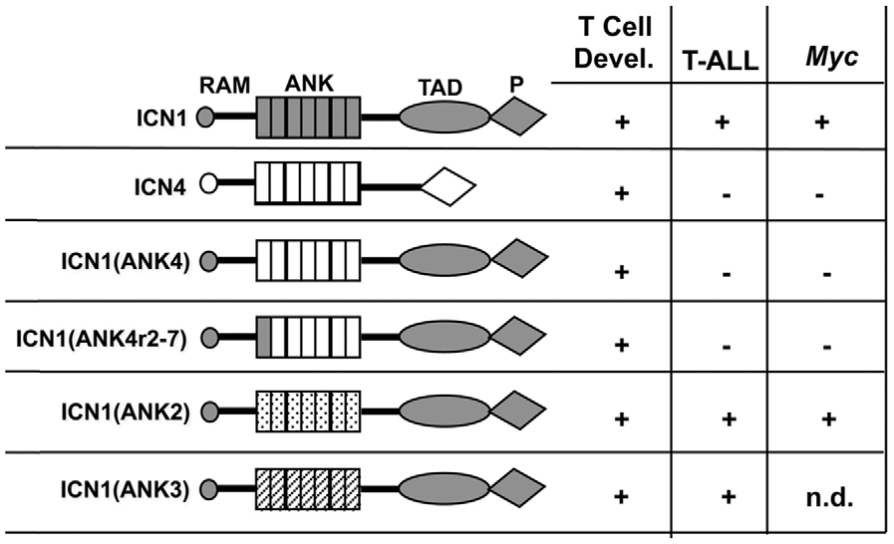
Summary of Effects of ICNs on Induction of T Cell Development, Induction of T-ALL, and *Myc* Transactivation. The domain organization of ICN chimeras is shown at left, and the ability of each to induce T cell development, induce T-ALL, and transactivate *Myc* is summarized at right. n.d. = not determined.

These studies indicate that ICN1-4 share the capacity to drive T cell development, but differ in leukemogenic potential. Specifically, ICN4 has a more limited propensity to cause T-ALL or support T-ALL cell growth, despite its ability to transform cells of other lineages, such as breast epithelial cells. Based on domain swaps, we find that this difference in outcomes stems primarily from variation in the highly conserved ANK domains, which appears to selectively influence the transactivation of various target genes. Our findings (summarized in Figure 7) provide strong evidence for subtle but significant functional divergence in the intracellular domains of mammalian Notch receptors.

The leukemogenicity of various ICN isoforms is correlated with their ability to transactivate *Myc*, a direct Notch target gene in T-ALL cells [39], [40], [41]. Myc provides important leukemogenic signals, as ectopic *Myc* expression rescues growth and survival of some, but not all, Notch-dependent human T-ALL cell lines when Notch signaling is inhibited [45], and conditional deletion of *Myc* impedes Notch1-dependent T-ALL development in mouse models [46]. Notch1 transactivation of *Myc* has also been implicated in the pathogenesis of other neoplasms, including murine mammary tumors [47].

Although several possible CSL/ICN binding sites have been identified near the proximal *Myc* promoter [39], [40], [41], additional work is needed to determine how Notch1 transactivates *Myc* and how ANK sequence variation influences this process. Some Notch target genes are regulated by a sequence-paired-site (SPS), a special “head to head” configuration of CSL binding sites that permits the formation of dimeric CSL/ICN/MAML complexes via interactions involving ANK [48]. Notably, dimerization defective ICN1 mutants induce T cell development, but cannot transactivate *Myc*, induce T-ALL or maintain T-ALL cell growth [49], a phenotype resembling that produced by ICN4. Thus, one possibility is that ICN4 fails to form functional dimeric transcription complexes on a yet-to-be-discovered SPS that governs *Myc* expression.

Three key residues involved in dimer contacts in ICN1 are K1946, E1950, and R1985 [48]. These residues vary slightly among the mammalian ICNs; for example, ICN4 has an arginine residue instead of a lysine residue at the position analogous to K1946 in ICN1. While studies in purified systems have not uncovered substantial differences in the ability of ICN1-4 to form complexes with CSL and MAML1 on DNA sequences containing single CSL sites or high affinity SPSs [48], [50], ICN4 might be unable to form dimers on low-affinity, non-consensus, “cryptic” SPSs, which have recently been identified near several direct Notch1 target genes [48]. On the other hand, however, in 8946 cells the ICN1(ANK4) chimeric polypeptide transactivated *Ptcra* (Figure S5), a dimerization-dependent Notch target that appears to be regulated by a cryptic SPS [49]. A second possibility is that ICN4 is fully competent for dimer formation, but unable to recruit additional unknown factors that are essential for transactivation of a subset of Notch target genes, including *Myc*. Both ideas are in line with a structural comparison of ANK1-4 (Figure S3), which shows that the most divergent surface residues lie away from CSL/MAML contacts on the backside of the ANK domains. This more divergent ANK surface participates in dimerization and could also be involved in differential recruitment of co-factors to Notch transcription complexes. Testing of these possibilities awaits further characterization of the genetic elements involved in Notch regulation of *Myc* and the biochemical composition of Notch transcription complexes.

The Notch pathway is an attractive therapeutic target in T-ALL [51], [52], but attempts to treat relapsed/refractory disease have revealed that non-selective GSIs cause “on-Notch” toxicity by skewing the differentiation of epithelial progenitors towards goblet cell fate. The ability of Notch to function as a tumor suppressor raises additional concerns about long-term toxicity associated with Notch pathway blockade. A detailed biochemical comparison of complexes formed by different Notch receptors (e.g., ICN1 and ICN4) may reveal opportunities for development of therapies that selectively interfere with the transactivation of subsets of Notch target genes within particular cellular contexts, thereby obviating toxicities associated with pan-Notch inhibitors.

## Materials and Methods

### Expression Plasmids

A cartoon depicting ICN1-4 is shown in Figure 1. The constructs encoding ICN1 (amino acids 1759 to 2556) and ICN2 (amino acids 1721 to 2471) have been described [53] The cDNA encoding ICN3 (amino acids 1667 to 2318) was the kind gift of Dr. Urban Lendahl. The ICN4 cDNA was created by PCR amplification of a lambda phage gt11 human umbilical vein endothelial cell cDNA library (the kind gift of Dr. David Ginsburg) with ICN4-specific primers, and encodes amino acids 1472 to 2003 of human Notch4. Each cDNA was engineered to have an ATG start codon within the same Kozak consensus sequence. These cDNAs were cloned into the plasmid pcDNA3 (Invitrogen) and the retroviral vector MigRI, which also contains an internal ribosomal entry sequence and a cDNA encoding GFP. Chimeric ICN cDNAs were created by introducing restriction sites by PCR that permitted the ligation of cDNA fragments of interest followed by site directed mutagenesis (QuikChange Kit, Stratagene). The identities of all expression constructs were confirmed by DNA sequencing. The specific points of joining that were used to create chimeric ICNs and the alignment of the ICN1-4 RAM and ANK domains are shown in Figure S1.

### Cell Culture

Cell culture reagents were obtained from Invitrogen. U2OS and 293T cells were obtained from the American Type Culture Collection. U2OS, 293T, and Bosc23 cells were grown in Dulbecco's modified Eagle's medium (DMEM) supplemented with 10% fetal bovine serum, 2 mM L-glutamine, 100 U of penicillin/ml, and 100 µg of streptomycin/ml. T6E cells and 8946 cells [39] were grown in RPMI 1610 media with the same additions. Cell lines were grown at 37°C under 5% CO_2_.

### Thymic Organ Culture

Harvest of fetal liver hematopoietic progenitor cells and seeding of thymic organ cultures were as described [54]. Briefly, liver cells from day 15 B6 fetal mice were isolated on a Ficoll gradient, washed with PBS, and suspended at 1–2×10^6^ cells/ml in 2 ml of Iscove Modified Dulbecco's medium containing 10% fetal calf serum, 2 mM glutamine, 0.5 mM penicillin and streptomycin, 50 ng/ml stem cell factor, 6 ng/ml interleukin-3, 4 ng/ml interleukin-1beta, and 1 ng/ml interferon-gamma. The following day, the cells were transduced with MigRI retroviruses and used to seed irradiated day 15 fetal thymic lobes (1×10^5^ cells/lobe) harvested from the same fetuses in hanging drops. After 24 hr, reconstituted fetal thymic lobes were placed on 0.8micron polycarbonate membranes (Isopore) in the presence of absence of 1 microM compound E (Tocris), a potent gamma-secretase inhibitor.

### Murine Bone Marrow Transplantation

Animal care and experimental protocols were performed according to guidelines from the National Institutes of Health with an approved protocol (801264) from the Institutional Animal Care and Use Committee at the University of Pennsylvania School of Medicine. Mouse experiments were performed in the BRB II/III animal facility at the University of Pennsylvania, which is staffed by animal handlers that are experienced in the care of rodents. Veterinarian services are available at the University of Pennsylvania. A veterinarian reviews all IACUC protocols prior to initiation of the study. The University Laboratory Animal Resources and Department of Laboratory Animal Medicine veterinarian makes regular inspections and provides veterinary care. Every effort was made to reduce the level of pain or discomfort in our studies. Mice were observed daily and when abnormal conditions/behavior was observed, it was reviewed with the attending veterinarian to determine the proper course of action. When euthanasia was required, it was accomplished according to the IACUC guidelines by exposure to carbon dioxide, consistent with the recommendations of the Panel on Euthanasia of the American Veterinary Medical Association.

Briefly, MigRI plasmids were packaged into ecotropic retroviruses by transient transfection of Bosc23 cells. Retroviral supernatants were titered on NIH 3T3 cells using GFP as a marker of transduction. Bone marrow cells from 5-fluoruracil-treated female 4–8 week old C57BL/6 mice (Taconic Farms) were transduced with GFP-normalized retroviral supernatants in DMEM containing 10% heat-inactivated FBS (Invitrogen), 5% WEHI-conditioned medium, 6 U/ml recombinant mouse IL-3 (Genzyme Corp., Cambridge, MA), 10,000 U/ml recombinant murine IL-6 (Genzyme), 5 U/ml recombinant murine stem cell factor (Genzyme), 4 microgram/ml polybrene (Sigma Chemical Co., St. Louis, MO), 100 U/ml streptomycin (Invitrogen), 100 U/ml penicillin (Invitrogen), and 2 mM L-glutamine (Invitrogen). Transduced bone marrow cells were injected into lethally irradiated (900 rads) 4- to 8-week-old female syngeneic recipients.

### Flow Cytometry

Peripheral blood samples and tumor cell suspensions were assessed for GFP^+^ immature T cells by flow cytometric analysis (Becton Dickinson, FACSCalibur). Cells were incubated with PE-labeled anti-CD8alpha (53–6.7), biotinylated anti-TCRβH57-597), and APC-labeled anti-CD4 (RM4-5) antibodies (Pharmingen). Biotinylated antibodies were revealed with streptavidin-PerCP. Dead cells, identified by forward and side scatter, were excluded from the analysis. FACS results were analyzed using CellQuest software.

### Cell Cycle Analyses

Analyses were carried out as described [39]. Live or fixed cells were stained with Hoechst 33342 (4 microM, Sigma B2261) or propidium iodide (40 microgram/ml), respectively, and then analyzed for DNA content and GFP expression by flow cytometry. Data analysis was performed using FlowJo software (Treestar).

### Reporter Gene Assays

Notch1 expression plasmids were introduced into U2OS cells by transient transfection with Lipofectamine Plus (Invitrogen) and assessed for their ability to activate an artificial Notch-sensitive reporter gene as described [18]. Briefly, cells in 24-well dishes were co-transfected with 1 ng or 10 ng of various pcDNA3-Notch expression constructs, a Notch-sensitive firefly luciferase reporter gene, and an internal control pRL-TK *Renilla* luciferase gene (Promega). Total transfected DNA was kept constant by adding empty pcDNA3 plasmid. Normalized firefly luciferase activities were measured in whole cell extracts prepared 44–48 hr after transfection using the Dual Luciferase kit (Promega) and a specially configured luminometer (Turner Systems).

### Protein purification

ANK1-4 polypeptides were expressed and purified as described [55]. BL21 (DE3) pLysS *Escherichia coli* were transformed with the appropriate plasmids and grown at 37°C in LB medium with 100 µg/mL ampicillin and 34 µg/mL chloramphenicol to an OD600 of 0.7–0.9. Protein expression was induced with 1 mM isopropyl-1-thio-β-D-galactopyranoside (IPTG) for 4 hr, and cells were harvested by centrifugation at 3500 rcf for 30 minutes. Pellets were frozen at −80°C after resuspension in 20 mL lysis buffer (50 mM Tris pH 8.0, 300 mM NaCl) per liter culture. After thawing in the presence of protease inhibitors (Complete, Roche), 5 mM β-mercaptoethanol, and 1 mM EDTA, cells were lysed by sonication and the clarified lysate was applied to glutathione-agarose beads (Sigma-Aldrich). Beads were washed with buffer A (50 mM Tris, pH 8.0, 150 mM NaCl, 5 mM DTT) and incubated with approximately 200 ng of tobacco etch virus (TEV) protease per 3 mL pelleted beads for 12–16 hr at room temperature. Following removal of the protease by application of the supernatant to Ni-NTA resin (Qiagen), ANK molecules were further purified by anion-exchange chromatography on a MonoQ 10/100 GL column using a linear gradient from 0.1–0.6 M NaCl in 20 mM Tris, pH 8.0, 5 mM DTT, 1 mM EDTA and size-exclusion chromatography on a Superdex S200 16/60 column in 20 mM Tris pH 7.5, 150 mM NaCl, 5 mM DTT. Proteins were concentrated to 5–10 mg/mL, aliquoted, flash frozen in liquid nitrogen, and stored at −80°C.

### Circular Dichroism Spectrocopy

CD spectra were acquired on an AVIV 62DS spectropolarimeter with proteins in 10 mM phosphate buffer at 5 to 10 µM for 1 mm path length cuvettes and 0.5–1 µM for 1 cm path length cuvettes. Concentrations were determined by absorbance at 280 nm [56]. Wavelength scans were performed at 4°C in a 1 mm path length cuvette using a 1 nm step size, a 3-second averaging time, and 5 repeats. Thermal melts were performed by monitoring the CD signal at 222 nm in a 1 cm path length cuvette and varying the temperature from 4°C to 80°C with a thermoelectric temperature controller, using a 2-minute equilibration period, a 1°C step size, and a 30-second acquisition time. Approximate melting temperatures were determined by fitting the thermal denaturation curves with the program IgorPro (Wavemetrics).

### Quantitative Reverse Transcriptase Polymerase Chain Reaction (qRT-PCR) Assays

Expression levels of the Notch target genes *Myc*, *Deltex1*, *Trb2*, *Notch3*, CD25, and *Ptcra* were determined by qRT-PCR as described [39], [57].

### Pathology

At necropsy, tissues were i) fixed in formalin and embedded in paraffin for histologic analysis, or ii) disaggregated for flow cytometry or injection into secondary recipients. Paraffin sections were stained with hematoxylin and eosin.

## Supporting Information

Figure S1 **Multi-sequence alignment of ICN1-4.** The aligned sequences correspond to the RAM and ANK domains of ICN1-4. Hydrophobic residues are colored red, hydrophilic residues are colored green, basic residues are colored magenta, and acidic residues are colored blue. AN and AC designate the N-terminal and C-terminal boundaries of the crystallized ANK domain of Notch1 (residues 1873–2127), while AS brackets the region of ANK that is structured (residues 1909–2123). J1 and J3 designate points of joining in chimeras in which entire ANK domains were swapped; J2 and J3 are points of ANK repeat 2–7 only swaps; while J3 is the point of joining in C-terminal sequences swaps. * = identical residues; : = conservative substitutions; . = semi-conservative substitutions.
(TIF)

Figure S2 **Circiular Dichroism Spectra and Thermal Stability of the Notch1-4 Ankyrin Repeats.** The four ANK molecules do not demonstrate heterogeneity in overall secondary structure characteristics. (A) Circular dichroism scans of the four domains at 4°C. (B) Temperature dependence of the CD signal at 222 nm for the four domains, with an apparent T_m_ between 46°C and 50°C for each ANK.
(TIF)

Figure S3 **Conservation of Surface Residues in the Notch1-4 Ankyrin Repeats.** The ANK domain of human Notch1 (pdb ID code 2F8Y) was rendered as a molecular surface using the program PyMol (DeLano Scientific). The inner concave and outer convex surfaces of the ANK domain are shown alone in the top two panels, and in the context of the ternary complex with MAML1 and CSL in the lower panels; “1” and “7” designate the amino- and carboxy-terminal ankyrin repeats 1 and 7, respectively. The ANK surface is colored according to sequence conservation among the ANK domains of Notch1-4 on a sliding scale from dark blue (100% conserved) to red (least conserved).
(TIF)

Figure S4 **ICNs have no Effect on 8946 Cell Growth when Transgenic** ***MYC*** **is “On”.** 8946 cells were transduced with empty MigRI or the indicated forms of ICN1 and monitored for growth in the presence of vehicle only (DMSO).
(TIF)

Figure S5 **Effect of Various ICNs on Notch Target Gene Expression in 8946 Cells.** 8946 cells transduced with the indicated MigRI viruses were sorted for GFP positivity, harvested after 24 hr of treatment with doxycycline (20 microgram/ml) or vehicle (PBS), and analyzed for expression of the various Notch target genes.
(TIF)
