# Supplementary figures and images for: Notch Ankyrin Repeat Domain Variation Influences Leukemogenesis and Myc Transactivation
Source: PLoS One. 2011 Oct 13;6(10):e25645. doi: 10.1371/journal.pone.0025645 (PMC3192765; doi:10.1371/journal.pone.0025645)

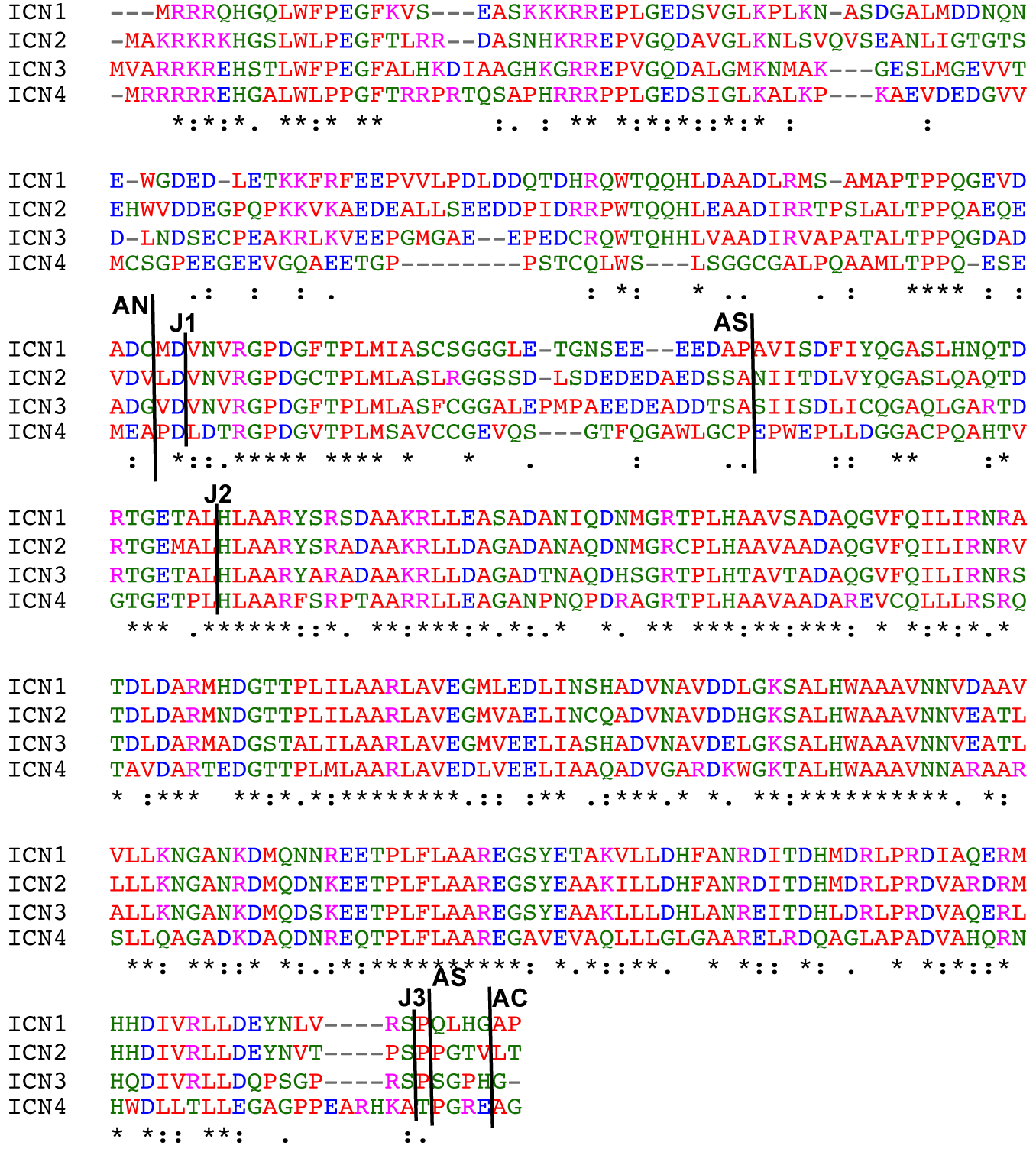

Supplement: Figure S1 — Multi-sequence alignment of ICN1-4. The aligned sequences correspond to the RAM and ANK domains of ICN1-4. Hydrophobic residues are colored red, hydrophilic residues are colored green, basic residues are colored magenta, and acidic residues are colored blue. AN and AC designate the N-terminal and C-terminal boundaries of the crystallized ANK domain of Notch1 (residues 1873–2127), while AS brackets the region of ANK that is structured (residues 1909–2123). J1 and J3 designate points of joining in chimeras in which entire ANK domains were swapped; J2 and J3 are points of ANK repeat 2–7 only swaps; while J3 is the point of joining in C-terminal sequences swaps. * = identical residues; : = conservative substitutions; . = semi-conservative substitutions. (TIF) [file pone.0025645.s001.tif]

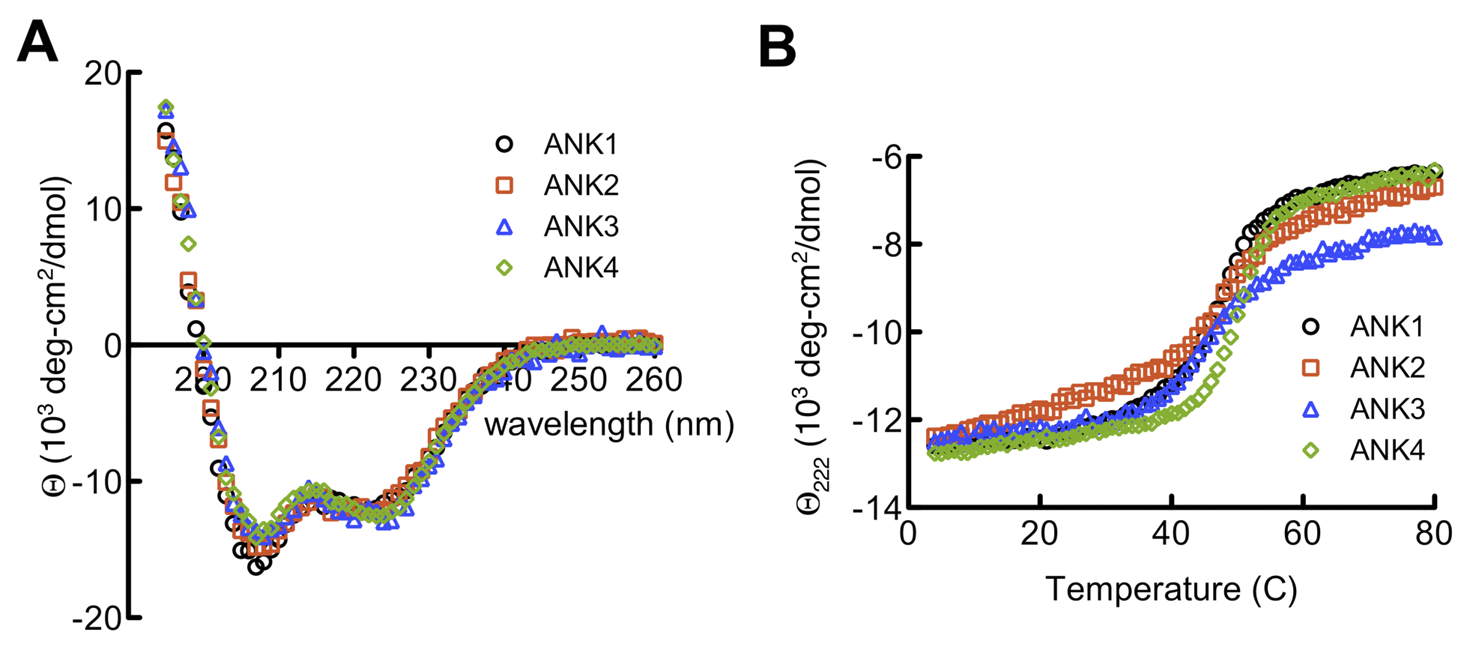

Supplement: Figure S2 — Circiular Dichroism Spectra and Thermal Stability of the Notch1-4 Ankyrin Repeats. The four ANK molecules do not demonstrate heterogeneity in overall secondary structure characteristics. (A) Circular dichroism scans of the four domains at 4°C. (B) Temperature dependence of the CD signal at 222 nm for the four domains, with an apparent Tm between 46°C and 50°C for each ANK. (TIF) [file pone.0025645.s002.tif]

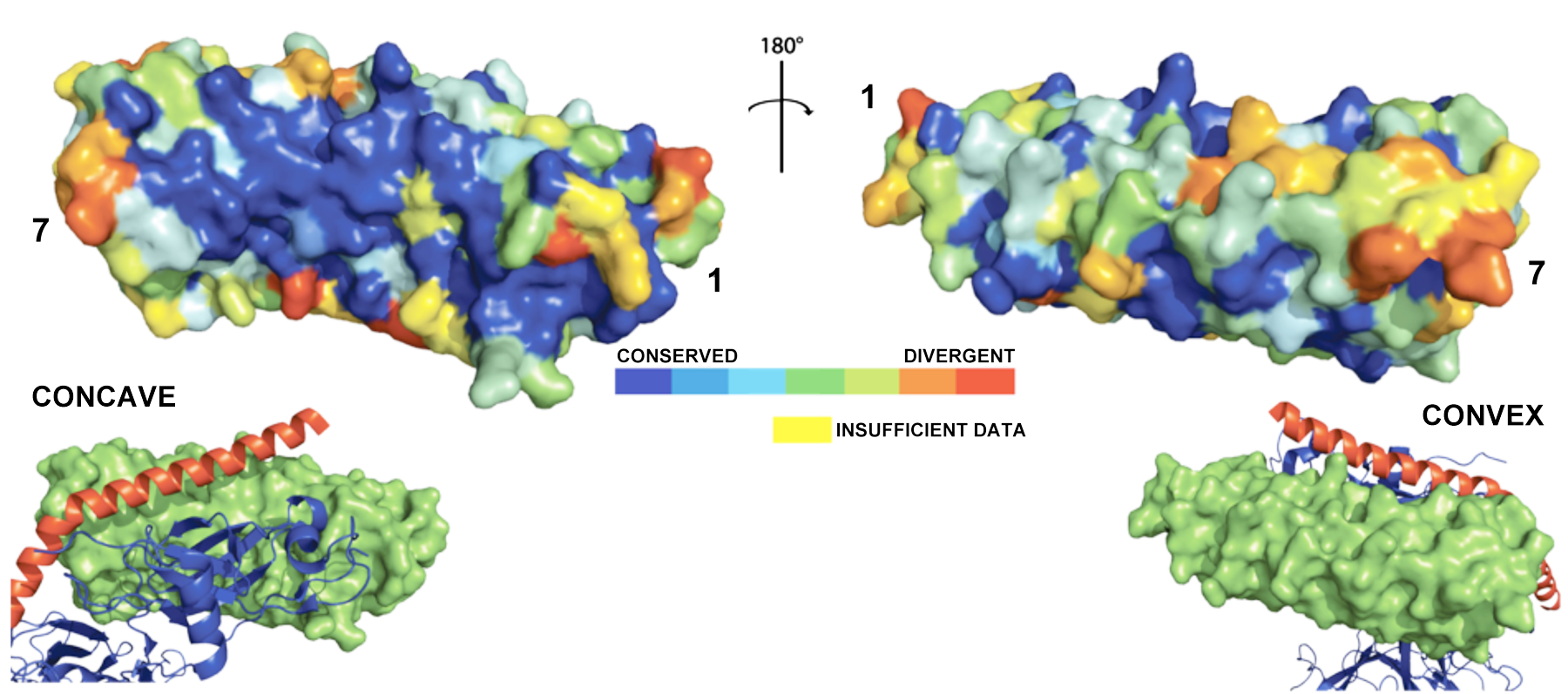

Supplement: Figure S3 — Conservation of Surface Residues in the Notch1-4 Ankyrin Repeats. The ANK domain of human Notch1 (pdb ID code 2F8Y) was rendered as a molecular surface using the program PyMol (DeLano Scientific). The inner concave and outer convex surfaces of the ANK domain are shown alone in the top two panels, and in the context of the ternary complex with MAML1 and CSL in the lower panels; “1” and “7” designate the amino- and carboxy-terminal ankyrin repeats 1 and 7, respectively. The ANK surface is colored according to sequence conservation among the ANK domains of Notch1-4 on a sliding scale from dark blue (100% conserved) to red (least conserved). (TIF) [file pone.0025645.s003.tif]

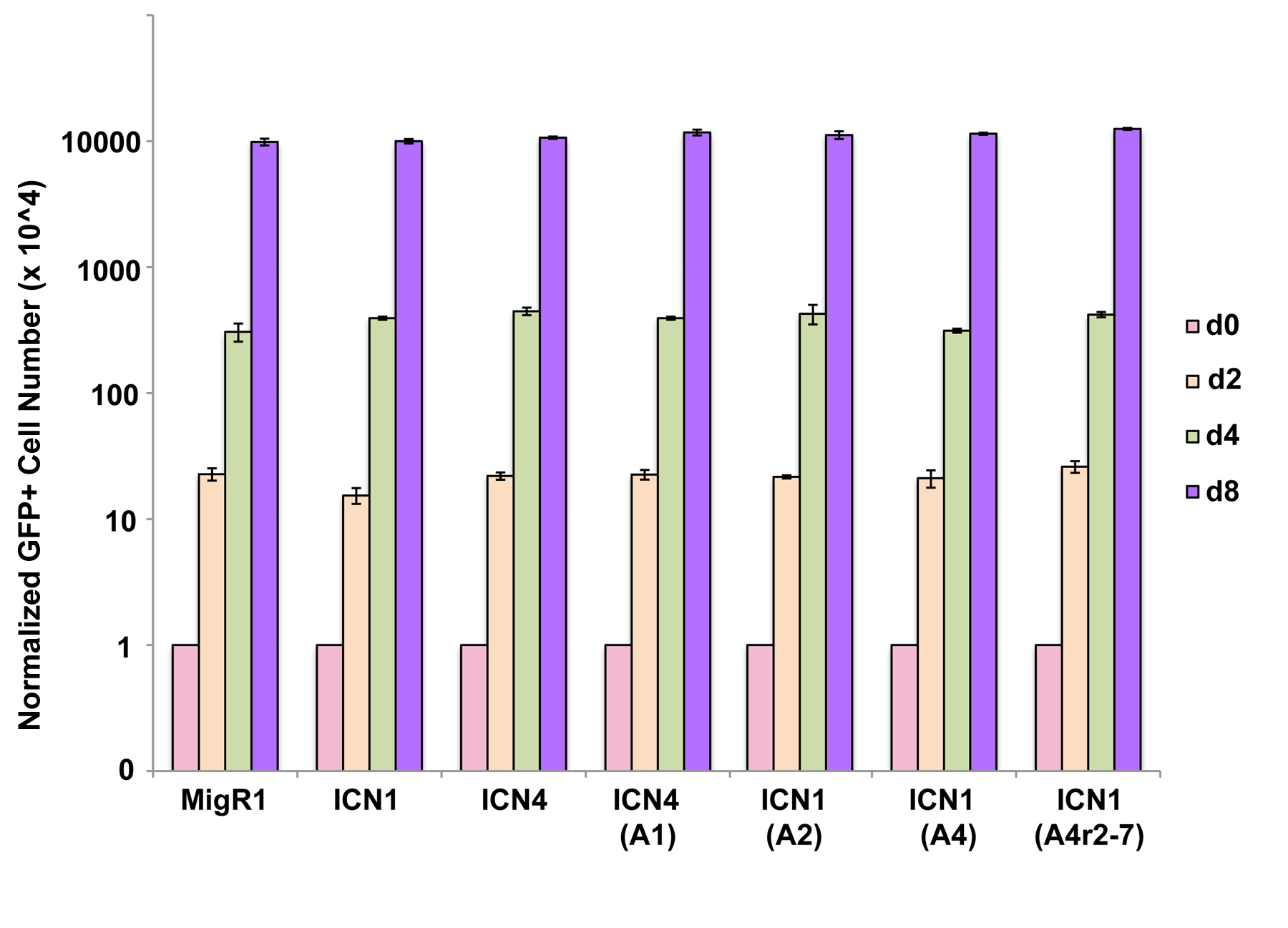

Supplement: Figure S4 — ICNs have no Effect on 8946 Cell Growth when Transgenic MYC is “On”. 8946 cells were transduced with empty MigRI or the indicated forms of ICN1 and monitored for growth in the presence of vehicle only (DMSO). (TIF) [file pone.0025645.s004.tif]

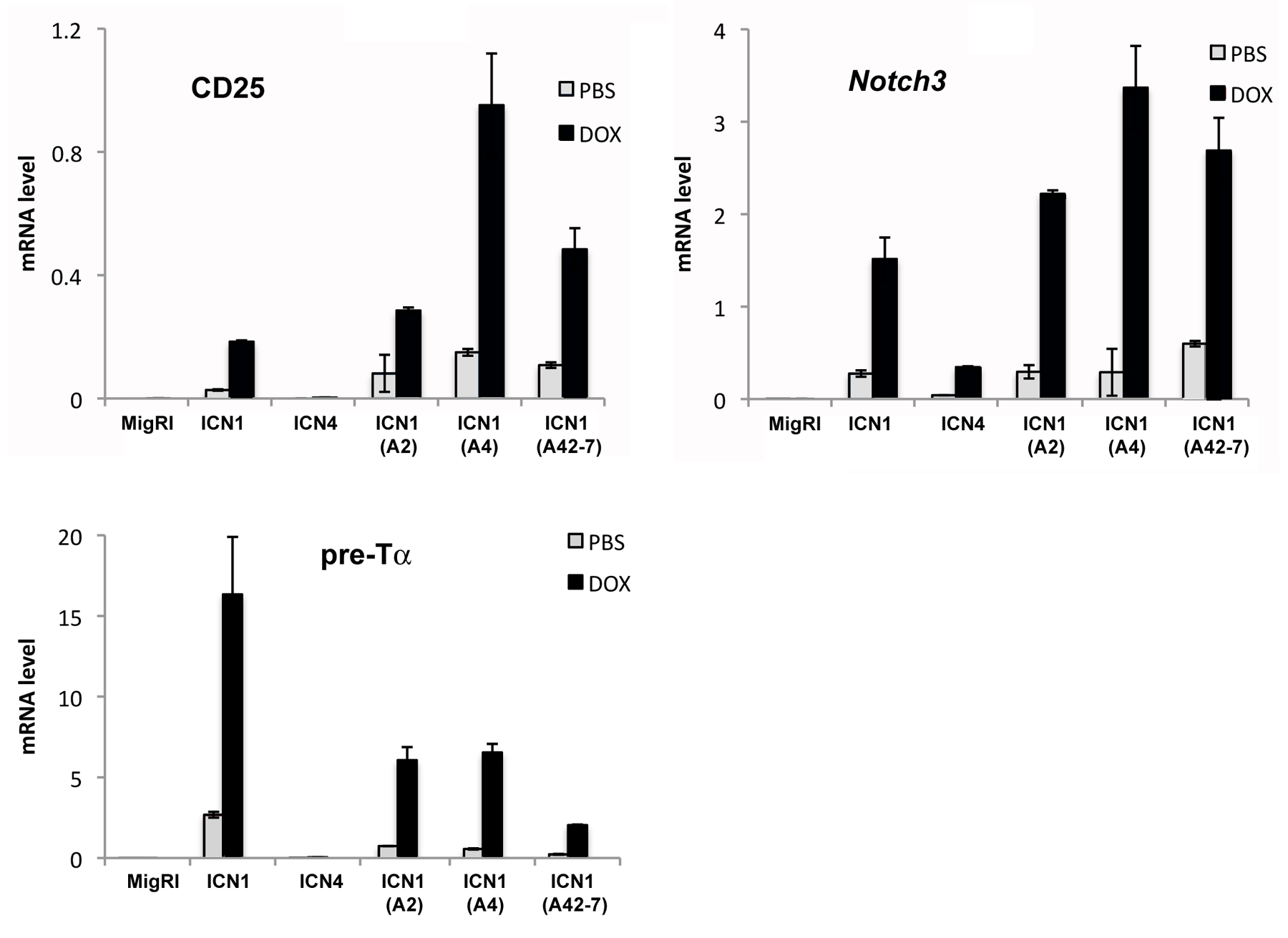

Supplement: Figure S5 — Effect of Various ICNs on Notch Target Gene Expression in 8946 Cells. 8946 cells transduced with the indicated MigRI viruses were sorted for GFP positivity, harvested after 24 hr of treatment with doxycycline (20 microgram/ml) or vehicle (PBS), and analyzed for expression of the various Notch target genes. (TIF) [file pone.0025645.s005.tif]
